# GLP-1 and GIP may play a role in long-term weight trajectories after gastric bypass

**DOI:** 10.3389/fendo.2025.1624001

**Published:** 2025-07-04

**Authors:** Sara Andrade, Carolina B. Lobato, Mariana Machado, Bolette Hartmann, Jens J. Holst, Rui F. Almeida, Mário Nora, Mariana P. Monteiro, Marta Guimarães, Sofia S. Pereira

**Affiliations:** ^1^ Unit for Multidisciplinary Research in Biomedicine (UMIB), School of Medicine and Biomedical Sciences (ICBAS), University of Porto, Porto, Portugal; ^2^ ITR-Laboratory of Integrative and Translational Research in Population Health, Porto, Portugal; ^3^ Department of Biomedical Sciences, Faculty of Health and Medical Sciences, University of Copenhagen, Copenhagen, Denmark; ^4^ Section of Endocrinology, Department of Medicine, Copenhagen University Hospital – Amager and Hvidovre, Hvidovre, Denmark; ^5^ Novo Nordisk Foundation Center for Basic Metabolic Research, Faculty of Health and Medical Sciences, University of Copenhagen, Copenhagen, Denmark; ^6^ Department of General Surgery, Hospital São Sebastião, Unidade Local de Saúde de Entre Douro e Vouga, Santa Maria da Feira, Portugal

**Keywords:** RYGB, suboptimal long-term outcomes, weight regain, T2D relapse, enteropancreatic hormones

## Abstract

**Introduction:**

Suboptimal clinical responses to metabolic and bariatric surgery include insufficient weight loss (WL), weight regain (WR), and/or comorbidity remission failure or relapse. Gut hormones’ role in WR and Type 2 diabetes (T2D) relapse is not fully established. So, our aim was to evaluate the hormone profiles of patients with long-term optimal and suboptimal response after gastric bypass (RYGB).

**Methods:**

This cross-sectional study included 43 individuals who underwent RYGB surgery over 10 years ago, divided into two groups: 23 participants with no T2D history but different WR trajectories (cohort 1), and 20 with prior T2D diagnosis and optimal WL (cohort 2), with post-RYGB T2D remission (n=10) or relapse (n=10).

**Results:**

Fasting and postprandial glucose, insulin, C-peptide, glucagon, GLP-1 and GIP levels were evaluated during a mixed-meal tolerance test. In cohort 1, fasting glucose, insulin, C-peptide, and glucagon, as well as the postprandial glucose and GIP levels, were significantly positively correlated with %WR. Additionally, postprandial GLP-1 and glucagon levels were negatively correlated with the %WR. In cohort 2, higher postprandial glucose and lower insulin were observed in participants with T2D relapse. No other significant differences were observed.

**Discussion:**

In sum, greater WR was associated with higher levels of postprandial glucose and GIP, along with lower GLP-1 and glucagon excursions. Whether these are cause or consequence of WR remains to be clarified. Additionally, GIP and GLP-1 profile of participants with T2D relapse did not differ from those with T2D remission.

## 1 Introduction

While Roux-en-Y gastric bypass (RYGB) leads to substantial and sustained weight loss (WL) in the majority of individuals, along with significant improvements or even resolution of obesity-related conditions, especially type 2 diabetes (T2D) (1), approximately 20–30% experience suboptimal clinical response within 10 years (1–3). These suboptimal outcomes include insufficient total weight loss (TWL <20% at 12 months), weight regain (WR) after initial weight nadir, and/or recurrence of T2D following an initial remission (4).

Although the mechanistic aspects of metabolic bariatric surgery (MBS), consisting in gastric volume restriction and intestinal malabsorption, were initially thought to be the only culprits for the observed response to MBS, it is long known that they are just part of the equation. Gut hormones also play a crucial role, as evidenced by the rapid normalization of blood glucose in individuals with T2D shortly after MBS, even before significant WL occurs (5, 6). Indeed, the mechanical modifications of the gastrointestinal tract in RYGB result in the earlier delivery of nutrients to the distal intestine that stimulate local neuro-endocrine cell populations, responsible for the secretion of several gut-derived hormones, such as Glucagon-like Peptide 1 (GLP-1) and Peptide YY (PYY), which contribute to increased satiety and improved glycemic control (7, 8). In fact, GLP-1 paramount role in mediating the effects of RYGB is now well established (5), and GLP-1 receptor agonists are used for obesity and diabetes treatment (9, 10). In contrast, the putative role of the other incretin hormone, Glucose-dependent Insulinotropic Polypeptide (GIP) in mediating the effects of MBS is much less clear given the disparities found in GIP levels after MBS and conflicting results from both human and animal studies (11). Therefore, it has been assumed that if there was any GIP contribution for MBS outcomes, it was likely to be negligible. Still, in non-operated euglycemic individuals, GIP seems to have a more important role in β-cell function than GLP-1 (5). Moreover, there is evidence that the two hormones have comparable activity after sleeve gastrectomy whereas GLP-1 stands out as predominant after RYGB (5), even though, the incretin effect of endogenous GIP is weak or absent in people with T2D (12). More recently, tirzepatide, a GLP-1 receptor and GIP receptor (GIPR) co-agonist, demonstrated greater WL and antidiabetic efficacy compared to isolated GLP-1 receptor (GLP-1R) agonists (13), GIP was brought back into the spotlight and elicited a renewed interest in further understanding its role in glucose and energy homeostasis (14, 15).

While MBS efficacy has been widely investigated, the reasons for a suboptimal clinical response, in particular whether gut hormones also play a role in WR or T2D relapse is less well established (8). Therefore, the aim of this study was to gain further insights into the putative mechanisms leading to MBS suboptimal response through the evaluation of hormone profiles of individuals with optimal and suboptimal response to MBS due to WR or T2D relapse.

## 2 Materials and methods

### 2.1 Participant selection

The study participants were identified from our retrospective cohort study that evaluated WL and comorbidity remission at 10 or more years after RYGB surgery (3, 16). A sub-set of these patients (N=43) were invited, accepted and gave their informed consent to join this study, which comprised two separate cohorts with different inclusion criteria.

#### 2.1.1 Cohort 1 – Analysis of hormone profiles of individuals with different weight trajectories (n=23)

To focus on weight trajectories analysis, we defined the inclusion criteria as individuals with no history of T2D who achieved optimal WL - defined as a TWL greater than 20% within two years after surgery. Exclusion criteria comprised individuals who had short- or long-term surgical complications or who were under drugs with glucose-lowering and/or obesity management medications.

Participants among this cohort included individuals who were able to sustain the WL and other individuals who experienced a suboptimal clinical response over the long term.

Given that there is lack of consensus on criteria for WR after a successful initial WL in patients submitted to MBS, participants were not grouped according to WL response. Instead, the %TWL and the percentage of WR after nadir were evaluated as continuous variables and correlated with the entero-pancreatic dynamics described below.

#### 2.1.2 Cohort 2 - Analysis of hormone profiles of individuals with sustained T2D remission vs T2D relapse (n=20)

To focus on T2D relapse analysis, we defined the inclusion criteria for this cohort as individuals diagnosed with T2D prior undergoing MBS with optimal WL over the long-term after surgery. Participants with short- or long-term surgical complications were excluded.

Participants were divided into two groups: participants who experienced either sustained T2D remission [HbA1c < 6.4% and FPG < 7.0 mmol/L without any antidiabetic medication for at least 3 months after surgery (17), (n=10)] or people with T2D relapse after remission (n=10), despite optimal WL over the long-term after surgery. These participants were matched by pre-operative age, pre-operative body mass index (BMI) and time since surgery. The participants were not taking drugs with glucose-lowering and/or weight-loss potential, except for the ones with T2D relapse, who were on metformin only.

This separate analysis of two different cohorts was designed to allow the identification of hormonal profiles related to WL trajectories and to T2D relapse, without co-interference.

The study was approved by the institutional ethical review board (approval numbers: CA-0172/19-0t_MP/AC and CA-149/2020-0t_MP/AC). All procedures performed were in accordance with the ethical standards of the institutional and national research committee and complied with the 1964 Helsinki declaration and its later amendments. Data are protected according to GDPR policies in place.

### 2.2 Surgical procedures

All surgeries were performed at a single bariatric surgery center, by the same team of general surgeons, using a standard laparoscopic RYGB technique with a fixed 120-cm alimentary limb and a differing biliopancreatic limb length: between 60 cm and 90 cm for the classical procedure or 200cm for the metabolic variant, as previously described (18). Participants were submitted to either surgical technique in a non-random assignment over 10 years earlier, according to the subjects’ anatomical and clinical features. Participants with no past medical history of T2D underwent a RYGB with a biliopancreatic limb length up to 90 cm long (classic RYGB), while participants with T2D diagnosed prior to surgery and optimal WL after RYGB who experienced either sustained T2D remission or T2D relapse, there was a balanced proportion of patients either submitted to classic (80% and 60%, respectively) or long (20% and 40%, respectively) biliopancreatic limb RYGB.

### 2.3 Mixed meal tolerance test

Participants underwent a mixed meal tolerance test (MMTT) following a 12 hour overnight fast. In addition, participants on metformin (cohort 2: participants with T2D relapse) took their last dose at least 12 hours before the MMTT.

Participants ingested a standardized commercially available liquid meal (Fresubin Energy Drink, 200 mL, 300 kcal [50E% carbohydrate, 15E% protein and 35E% fat]; Fresenius Kabi Deutschland, Bad Homburg, Germany) over a maximum period of 15 minutes. Venous blood samples were collected into EDTA tubes (S-Monovette^®^ 7.5 ml, K2 EDTA Gel, 1.6 mg/mL, Sarstedt), before the meal (−15 and 0 min) and at 15, 30, 45, 60, 90, and 120 min after the start of meal intake. The plasma was then separated and stored at −20°C until assayed.

### 2.4 Biochemical measurements

Blood glucose was measured at every timepoint of the MMTT using a glucometer (Freestyle Precision Neo Glucose meter, Abbott, USA). Insulin and C-peptide levels were measured by an electrochemiluminescence sandwich immunoassay (ECLIA) on Atellica^®^ (Siemens), as previously described (19). The lower detection limits for insulin and C-peptide were 4.8 pmol/L and 36.4 pmol/L, respectively. Inter- and intra-assay coefficients of variation were below 5%.

For GIP, GLP-1 and glucagon measurements, plasma samples were extracted using 70% ethanol. Total GLP-1, total GIP, and glucagon levels were measured using validated in-house radioimmunoassays (RIA) targeted to the C-terminal of GLP-1 (antiserum 89390), GIP (antiserum 867) and glucagon (antiserum 4305) (20). The lower detection limits for GLP-1 and GIP were 5 pmol/L, and 2.5 pmol/L for glucagon. Average inter- and intra-assay coefficients of variation were below 15%.

### 2.5 Calculations and statistical analysis

The % TWL [(preoperative weight – weight at MMTT) ÷ (preoperative weight) x 100], and the %WR [(weight at MMTT – nadir weight) ÷ (nadir weight) x 100] were determined.

HOMA Calculator version 2.2.4 (http://www.dtu.ox.ac.uk, accessed November 2023) was used to determine the updated homeostasis model assessment indexes (HOMA2) as surrogate measures of beta cell function (HOMA2-B), peripheral insulin sensitivity (HOMA2-S) and resistance (HOMA2-IR).

Total and incremental areas under the curve (tAUC and iAUC, respectively) were calculated using the trapezoidal rule, without and with subtraction of the fasting hormonal levels from the subsequent timepoints, respectively. The fasting values were assumed as the mean value of the two fasting samples. The insulinogenic index (IGI) was calculated as the ratio of the incremental C-peptide from fasting to 30 minutes during the MMTT to glucose changes within the same period. The oral glucose insulin sensitivity (OGIS) was assessed and then multiplied by IGI to compute the Disposition Index, which targets insulin secretion corrected for insulin sensitivity. Insulin secretion rate (ISR) was determined by deconvolution from C-peptide plasma levels (CV 5%) with correction for age, sex and BMI using the ISEC program (ISEC, Version 3.4a, Hovorka, 1994). Finally, insulin clearance was calculated as the ratio between tAUC of the ISR and the tAUC of insulin.

Since participants in Cohorts 1 and 2 exhibited distinct pre- and post-operative characteristics, and the study aims to separately assess outcomes related to WL and T2D remission/relapse, no statistical comparisons were made between the two cohorts. So, all the statistical analyses were performed within each cohort.Nominal variables were expressed as number of cases and percentage (%), and the continuous variables are expressed as mean ± standard deviation (SD), unless stated otherwise. Missing data was handled by complete-case analysis, excluding participants with missing data for the variables analyzed. For nominal variables, the Fisher’s exact test was used. Continuous variables that did not meet the assumption of normality (Cohort 2: follow-up time; T2D duration; HbA1c at MMTT; fasting glucose; insulin clearance; IGI, disposition index; ISR iAUC; C-peptide tAUC; insulin iAUC and tAUC; GLP-1 tAUC) were analyzed using the Mann-Whitney test. The remaining variables met the normality assumption and were analyzed using unpaired two-tailed t-tests. Comparisons between timepoints during the MMTT were performed using a two-way analysis of variance (ANOVA) with Sidak’s *post hoc* test, for cohort 2. Linear regression was performed to assess the predictive value of preoperative age, weight and BMI for %TWL and %WR 10 years after surgery.

WR and TWL correlation with glucose and hormonal profiles were evaluated using the Pearson correlation test. A *post hoc* power analysis for sample size was performed for these correlations, with our primary hypothesis being that postprandial GLP-1 levels would be inversely correlated with %WR. This hypothesis was based on the well-established effect of GLP-1 on WL following RYGB (21). A power of 80.8% was achieved with a sample size of n = 23 using a one-sided test (as an inverse correlation was hypothesized), with an r-value cutoff of -0.50 and a significance level of p < 0.05.

Statistical analyses were performed using the GraphPad Prism version 10.1.1 and the IBM SPSS statistics software version 20.0.0.0. A p<0.05 was considered statistically significant.

## 3 Results

### 3.1 Anthropometric and glycemic status trajectories after RYGB surgery

Study participants’ characteristics before RYGB and at MMTT are presented in **Table 1**. In the cohort with no history of T2D (Cohort 1), the %TWL since surgery up to MMTT ranged between 0% and 43.22% while %WR after nadir ranged from 0% to 47.06% (**Supplementary Figure 1**). Using linear regression, we found that pre-operative BMI significantly predicted the %TWL after surgery (β= -1.71 [95% CI -2.95; -0.46], p=0.009) and the %WR after nadir (β= 6.02 [95% CI 1.47; 10.6], p=0.012) (**Supplementary Table 1**).

**Table 1:** Anthropometric and glycemic status trajectories after RYGB.

	Cohort 1: no prior T2D	Cohort 2: pre-operative T2D		
		Remission	Relapse	p
N	23	10	10	—
Sex, m:f (%)	4:19 (17.4%:82.61%)	1:9 (10.0%:90.0%)	0/10 (0.0%:100.0%)	>0.999
Type of RYGB, classic:metabolic (%)	0:23 (0.0%:100.0%)	8:2 (80.0%:20.0%)	6:4 (60.0%:40.0%)	0.629
Pre-operative				
Age (years)	34.13 ± 6.06	47.10 ± 8.74	49.40 ± 5.74	0.495
Body weight (kg)	120.20 ± 13.56	113.5 ± 12.1	105.3 ± 14.8	0.192
BMI (kg/m^2^)	45.36 ± 3.68	45.38 ± 5.31	42.96 ± 5,04	0.309
Post-operative				
Age (years)	48.35 ± 6.29	62.10± 7.98	62.90 ± 5.90	0.802
Body weight (kg)	93.39 ± 21.75	77.0 ± 8.9	72.4 ± 11.1	0.320
BMI (kg/m^2^)	35.22 ± 7.26	30.85 ± 4.40	29.61 ± 4.57	0.544
Time since surgery (years)	12.30 ± 1.02	13.00 ± 1.41	12.80 ± 0.92	0.739
Body weight nadir (kg)	76.91 ± 13.64	72.10 ± 8.10	69.55 ± 10.5	0.552
TWL nadir (%)	36.25 ± 5.87	36.35 ± 5.13	33.89 ± 4.62	0.274
TWL MMTT (%)	22.84 ± 11.87	31.98 ± 5.93	31.03 ± 7.18	0.750
WR (%)	20.91 ± 2.92	8.43 ± 7.22	6.37 ± 6.02	0.496
T2D duration before RYBG (years)	NA	3.25 ± 3.77	5.00 ± 2.60	0.067
FPG MMTT (mg/dl)	77.00 ± 3.70	80.50 ± 14.76	130.10 ± 40.10	**0.005**
HbA1c MMTT (%)	5.30 ± 0.28	5.58 ± 0.33	6.91 ± 0.93	**0.002**

Data is presented as mean ± standard deviation (SD)*. T2D* type 2 diabetes, *RYGB* Roux-en-Y gastric bypass, *BMI* body mass index, *TWL* total weight loss, *MMTT* mixed meal tolerance test, *NA* Not applicable, *WR* weight regain, *FPG* fasting plasma glucose, *HbA1c* glycated hemoglobin. Only the groups within Cohort 2 were compared*. Unpaired t-test or Mann-Whitney test (depending on the variables normality): statistically significant differences (p<0.05) are highlighted in bold.*

Participants with T2D before RYGB (Cohort 2) experienced remission in the first post-operative year, with the sole exception of one participant (who underwent T2D remission 5 years after RYGB). Among those who relapsed, this was observed 7.22 ± 4.18 years after surgery. No significant differences in post-operative body weight, BMI and %TWL at time of evaluation were observed when participant subgroups with sustained T2D remission *vs* relapse were compared nor correlated with pre-operative BMI (**Table 1**). Before surgery, none of the patients were under insulin therapy. However, patients with T2D relapse had higher HbA1c levels (5.47 ± 0.43 vs 7.08 ± 0.63, p=0.045) and longer T2D duration before RYBG (3.25 ± 3.77 vs 5.00 ± 2.60, p=0.067).

### 3.2 Glucose, insulin and C-peptide profiles

**Figure 1:**
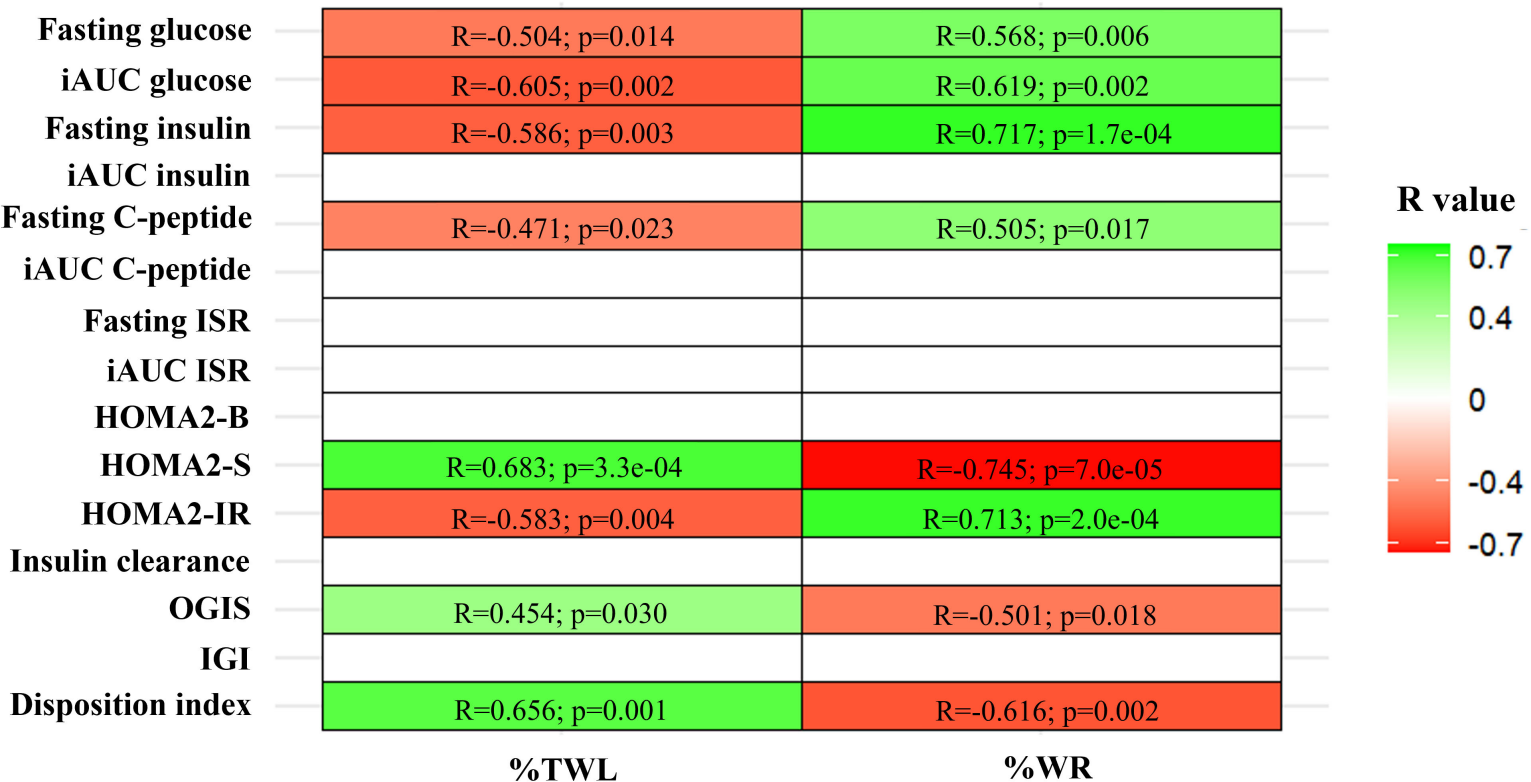
Correlations between % total weight loss (%TWL) and % weight regain (%WR) with fasting and postprandial (iAUC) levels of glucose, insulin, and C-peptide, as well as insulin secretion rate (ISR), markers of beta-cell function (HOMA2-B), peripheral insulin sensitivity (HOMA2-S), insulin resistance (HOMA2-IR), insulin clearance, oral glucose insulin sensitivity (OGIS), insulinogenic index (IGI), and disposition index. Only patients with no Type 2 diabetes history were included (Cohort 1, n=23). *Pearson correlation test: colored cells indicate statistically significant positive (green) or negative (red) correlations between the variables*.

In participants without previous history of T2D (Cohort 1), fasting glucose, insulin, and C-peptide levels, along with insulin resistance (HOMA2-IR), were negatively correlated with %TWL. In contrast, insulin sensitivity (HOMA2-S and OGIS) and the disposition index were positively correlated with %TWL (**Figure 1**). The opposite correlations were observed when these were analyzed against the %WR (**Figure 1**).

**Figure 2:**
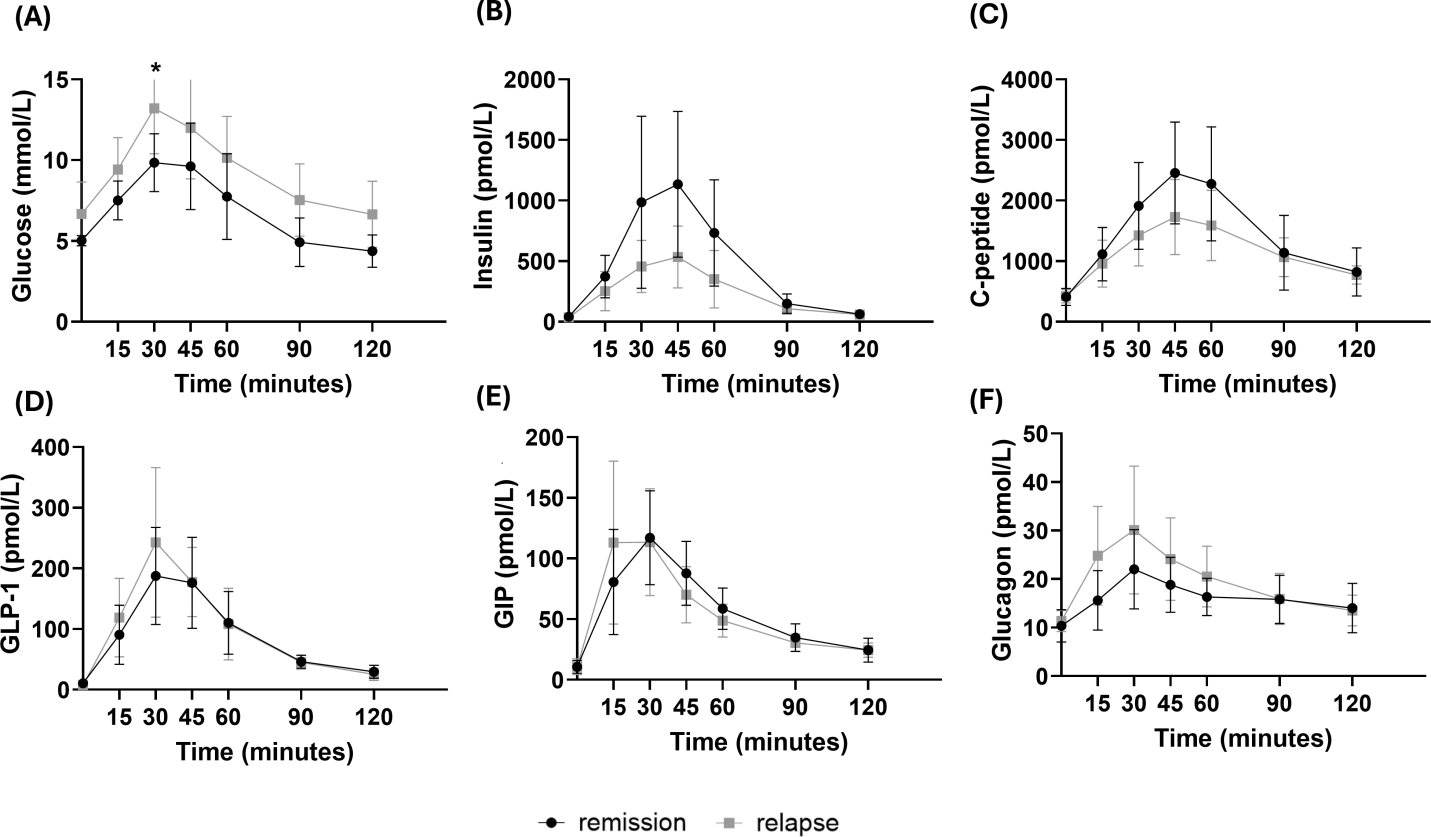
Peripheral levels of glucose **(A)**, insulin **(B)**, C-peptide **(C)**, glucagon-like peptide 1 [GLP-1, **(D)**], glucose-dependent insulinotropic polypeptide [GIP, **(E)**] and Glucagon **(F)** in response to a mixed meal tolerance test in patients with Type 2 diabetes remission or relapse after remission (Cohort 2, n=10 *per* group). Data is presented as mean ± SD. *Two-way ANOVA with Sidak’s post hoc test: No significant differences observed*.

In the cohort of participants with T2D prior to RYGB, significantly greater postprandial glucose (tAUC 1123.0 ± 260.6 vs 829.4 ± 165.0 mmol/L x min, p= 0.008) (**Table 2**; **Figure 2**) and lower insulin (tAUC 30911 ± 13770 vs 59580 ± 26382 pmol/L x min, p=0.005) excursions were observed in the T2D relapse subgroup (*vs* remission), but no significant differences in C-peptide excursion profiles were observed between subgroups (**Table 2**). IGI was also lower in the subgroup with T2D relapse *vs* T2D remission (IGI: 0.7 ± 0.5 *vs* 1.9 ± 1.6, p=0.009) (**Table 2**). Moreover, significantly lower HOMA2-B (48.7 ± 25.3% *vs* 78.4 ± 20.3%, p=0.010) and OGIS (352.6 ± 69.4 *vs* 417.8 ± 35.9, p=0.017) were found in the T2D relapse subgroup as compared to the T2D remission one, while insulin clearance was significantly higher (0.020 ± 0.01 *vs* 0.014 ± 0.01, p=0.024) (**Table 2**).

**Table 2:** Glucose, insulin, C-peptide dynamics, as well as insulin secretion rates and sensitivity after RYGB.

	Cohort 1: no prior T2D	Cohort2: pre-operative T2D		
		remission	relapse	p
Glucose				
Fasting (mmol/L)	4.91 ± 0.83	5.02 ± 0.31	6.67 ± 1.98	**0.005**
iAUC (mmol/L x min)	202.27 ± 70.10	279.7 ± 113.5	341.2 ± 140.9	0.297
tAUC (mmol/L x min)	714.59 ± 199.37	829.4± 165.0	1123.0 ± 260.6	**0.008**
Insulin				
Fasting (pmol/L)	44 ± 34	40 ± 12	35 ± 12	0.378
iAUC (pmol/L x min)	34430 ± 13474	54741 ± 25762	26661 ± 13198	**0.007**
tAUC (pmol/L x min)	39747 ± 14854	59580 ± 26382	30911± 13770	**0.005**
C-peptide				
Fasting (pmol/L)	265 ± 119	408 ± 140	422 ± 110	0.819
iAUC (pmol/L x min)	52521 ± 7340	133904 ± 53403	93445 ± 36457	0.063
tAUC (pmol/L x min)	83867 ± 9368	182972 ± 65951	144085 ± 13432	0.248
ISR				
Fasting [pmol/(kg x min)]	0.7 ± 0.31	1.2 ± 0.4	1.3 ± 0.4	0.537
iAUC	199.0 ± 140.2	549.6 ± 232.6	397.1 ± 168.2	0.110
tAUC	259.3 ± 168.1	684.5 ± 276.4	555.6 ± 188.0	0.238
IGI	2.4 ± 2.0	1.9 ± 1.6	0.7 ± 0.5	**0.009**
Disposition Index	0.012 ± 0.012	0.009 ± 0.012	0.002 ± 0.004	0.132
HOMA2-B (%)	82.4 ± 21.5	78.4 ± 20.3	48.7 ± 25.3	**0.010**
HOMA2-IR	0.8 ± 0.7	0.8 ± 0.2	0.7 ± 0.2	0.579
HOMA2-S (%)	160.9 ± 70.6	143.9 ± 46.9	157.5 ± 51.4	0.542
OGIS	428.5 ± 63.5	417.8 ± 35.9	352.6 ± 69.4	**0.017**
Insulin Clearance	0.006 ± 0.003	0.014 ± 0.01	0.020 ± 0.01	**0.024**

Data is presented as mean ± SD. T2D type 2 diabetes, iAUC incremental area under the curve, tAUC total area under the curve, IGI insulinogenic index, HOMA2-B homeostasis model assessment for β-cell function, HOMA2-IR homeostasis model assessment for insulin resistance, HOMA2-S homeostasis model assessment for insulin sensitivity, OGIS oral glucose insulin sensitivity. Only the groups within Cohort 2 were compared. *Unpaired t-test or Mann-Whitney test (depending on the variables normality): statistically significant differences (p<0.05) are highlighted in bold.*

### 3.3 GLP-1, GIP and glucagon dynamics

**Figure 3:**
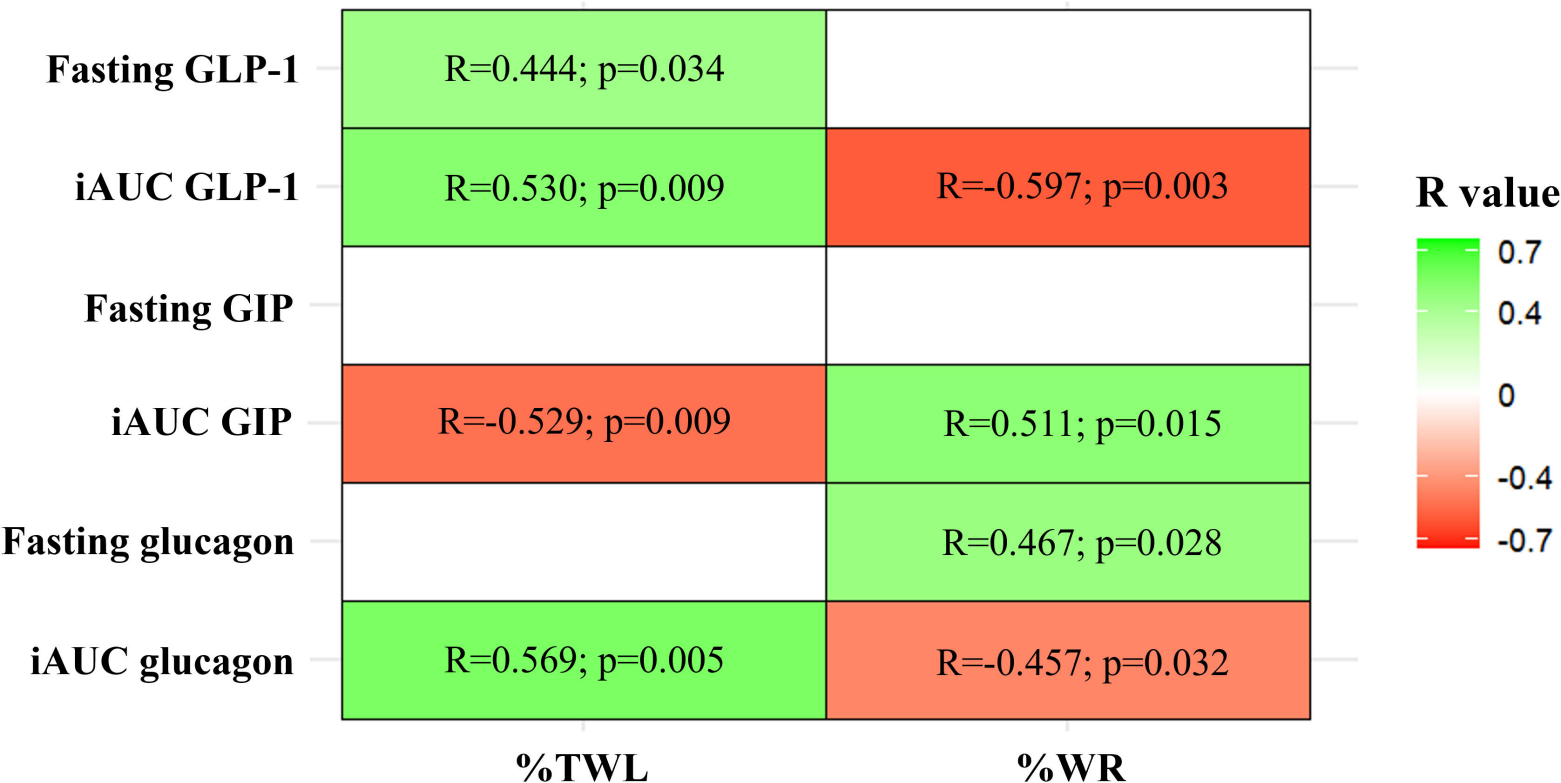
Correlations between % total weight loss (%TWL) and % weight regain (%WR) with fasting and postprandial (iAUC) levels of glucagon-like peptide 1 (GLP-1), glucose-dependent insulinotropic polypeptide (GIP) and glucagon. *Pearson correlation test: colored cells indicate statistically significant positive (green) or negative (red) correlations between the variables*.

In participants without a history of T2D, fasting GLP-1, postprandial GLP-1, and postprandial glucagon levels were positively correlated with %TWL after surgery, while postprandial GIP levels were negatively correlated with %TWL. In contrast, postprandial GLP-1 and glucagon levels were negatively correlated with %WR, whereas postprandial GIP levels and fasting glucagon levels were positively correlated with %WR (**Figure 3**).

Comparing the subgroups of individuals with T2D prior to surgery, no significant differences in fasting or postprandial excursion of GIP, GLP-1 and glucagon were observed (**Figure 2**; **Table 3**).

**Table 3:** Glucagon, GLP-1 and GIP dynamics after RYGB.

	Cohort 1: no prior T2D	Cohort 2: pre-operative T2D		
		Remission	Relapse	p
Glucagon				
Fasting (pmol/L)	10 ± 4	10 ± 3	11 ± 2	0.436
iAUC (pmol/L x min)	577 ± 263	720 ± 286	1058 ± 667	0.158
tAUC (pmol/L x min)	1687 ± 369	1962 ± 585	2411 ± 728	0.145
GLP-1				
Fasting (pmol/L)	9 ± 5	10 ± 8	7 ± 4	0.294
iAUC (pmol/L x min)	5979 ± 2168	9993 ± 3489	11421 ± 4399	0.432
tAUC (pmol/L x min)	7020 ± 2365	11195 ± 3797	12309 ± 4340	0.529
GIP				
Fasting (pmol/L)	11 ± 7	11 ± 5	11 ± 7	0.912
iAUC (pmol/L x min)	4997 ± 1798	5858 ± 644	5626 ± 744	0.817
tAUC (pmol/L x min)	6284 ± 1930	7088 ± 1790	6903 ± 2430	0.848

Data is presented as mean ± SD. T2D type 2 diabetes, iAUC incremental area under the curve, tAUC total area under the curve, GLP-1 glucagon-like peptide-1, GIP glucose-dependent insulinotropic polypeptide. Only the groups within Cohort 2 were compared. *Unpaired t-test or Mann-Whitney test (depending on the variables normality): statistically significant differences (p<0.05) are highlighted in bold.*

## 4 Discussion

This study shows that long-term WR after RYGB is associated with elevated fasting glucose, insulin resistance, and a blunted postprandial GLP-1 response alongside an exaggerated GIP response. On the other hand, T2D relapse correlates with impaired insulin secretion but not with altered incretin dynamics.

In particular, although the participants have no medical history of T2D, the patients with higher %WR have higher fasting levels of glucose, insulin, C-peptide and insulin resistance, as well as lower insulin sensitivity. The significant correlation between the %WR with glucose postprandial excursion but not with insulin/C-peptide postprandial profiles suggests that individuals with higher %WR face an additional challenge to control glucose levels after meals, unsurprisingly given the well-known association of obesity with increased risk of dysglycemia (22). Nonetheless, when corrected for insulin sensitivity (through calculation of the disposition index), there is an impairment of insulin secretion is those with more significant WR/lesser WL, which might possibly suggestive of beta-cell exhaustion, while perfect aligning with the documented relative lack of GLP-1 and glucagon postprandially, both of which known to be powerful insulinotropic hormones (23, 24).

We found significantly higher glucose and lower insulin excursions in the T2D relapse subgroup when compared to the T2D remission subgroup. Furthermore, lower HOMA2-B, OGIS and IGI were present in the T2D relapse subgroup. All together, these support the hypothesis of β-cell exhaustion at the end road of insulin resistance, consistent with the pathological mechanisms underlying T2D (25, 26). In addition, a significantly higher insulin clearance was observed in patients with T2D relapse compared to patients with T2D remission. This could be explained by the fact that when T2D relapses, as a consequence of inadequate insulin secretion, there is a rise in glucose levels in the bloodstream. In the presence of normal insulin sensitivity, this could lead to the upregulation of insulin receptor expression primarily in the liver, responsible for the majority of the uptake of insulin and therefore its clearance. Indeed, this phenomenon of increased insulin clearance has been previously reported in patients with early onset T2D diabetes and normal insulin sensitivity (27). When focusing on hormone profiles, participants with higher % of WR present lower levels of fasting and postprandial levels of GLP-1. One of the key mechanisms contributing to successful initial WL following bariatric surgery is GLP-1 mediated. Previous studies have consistently demonstrated that postprandial GLP-1 levels rise after RYGB (28) and that patients who experience greater WL after MBS have higher levels of GLP-1 compared to those with suboptimal WL (8, 29–32). In addition, and in line with our results, higher postprandial GLP-1 levels have also been observed in individuals with sustained WL, compared to those who experienced WR (32, 33). However, these results were not consistently confirmed by all studies reporting long-term outcomes. In particular, Lompropoulos et al. compared patients with successful WL maintenance and patients with WR 7 years post-surgery and found no significant differences in post-prandial GLP-1 levels (34).

In contrast to GLP-1 postprandial levels, which have been consistently reported to increase after RYGB, reports regarding the circulating levels of GIP following MBS are fewer and results have been inconsistent (28). Gao et al., in a meta-analysis, showed that changes in fasting GIP levels after RYGB are influenced by diabetes status. Specifically, individuals with T2D tended to show a more marked reduction in fasting GIP levels compared to those without T2D. In contrast, studies conducted in populations including both individuals with and without T2D often did not observe significant changes (35). In addition, short-term WL was not a significant predictor of fasting GIP reduction after RYGB (35). Sima E et al. also reported comparable levels of GIP during an oral glucose tolerance test between patients with and without optimal WL five years post-RYGB. On the other hand, Santo M et al. with a follow-up ranging from 27 to 59 months found that postprandial GIP levels were significantly different in patients with WR compared to those who experienced optimal WL maintenance (31, 32). The present study demonstrates that participants with greater WR after RYGB have higher GIP excursion following the intake of a mixed meal, despite having similar baseline levels. Nevertheless, it remains unclear whether these altered incretin dynamics are a cause or consequence of WR. It is possible that impaired GLP-1 signaling reduces satiety, leading to increased caloric intake and subsequent WR, while higher GIP levels may exacerbate this effect by promoting fat deposition (36, 37). Alternatively, WR itself might induce changes in incretin secretion. Both fasting and postprandial GLP-1 levels are lower in individuals with obesity compared to normal-weight (38), while GIP levels have been reported to be higher in people with obesity in both basal (39) and stimulated states (40). Therefore, the results related to GIP and GLP-1 excursion observed in our study could be simply explained by the differences in BMI between the groups. Noteworthy is the fact that, in our study, no significant correlations were observed between %WR and fasting GIP and GLP-1 levels, while it is the response to the mixed meal that is distinct, which suggests that the BMI is unlikely to be the sole reason for the differences observed. Diets with high fat content were also reported to elicit greater postprandial GIP excursions (41). However, in our study the meal tolerance test was performed with a standardized mixed meal and all subjects had exactly the same meal challenge.

The discrepancies in the results across studies regarding the relation between postprandial GIP and GLP-1 levels and WR may be attributed to differences in the criteria used to define suboptimal outcomes, as well as the inclusion of patients with T2D in some analyses. People with T2D are known to exhibit distinct gastrointestinal hormone profiles (42). To minimize this bias regarding weight trajectories, our study excluded participants with a history of T2D from the %WR analysis and defined TWL and WR as non-categorical continuous variables, without committing any artificial or arbitrary cut-offs. Additionally, the divergences that were observed may suggest the involvement of other mechanisms in long-term weight maintenance after MBS.

Individuals with higher %WR exhibited higher fasting glucagon levels, along with higher glucose and insulin levels, likely reflecting their impaired insulin sensitivity. However, a contrasting pattern emerged when analyzing postprandial glucagon levels, as patients with greater %WR showed lower postprandial glucagon levels. While glucagon had not previously been directly correlated with WR after MBS, previous studies suggested that glucagon may contribute to WL by suppression of appetite and activation of energy expenditure and thermogenesis (43). The discrepancy between fasting and postprandial glucagon levels correlation with %WR can be explained by the complex regulation of glucagon secretion and action after MBS (44).

There was an upsurge of GIP focused research since the extended-release GLP-1R and GIPR co-agonist tirzepatide became available for T2D and obesity treatment (13). However, the mechanism by which weight reduction is achieved is not totally clear, nor to what extent is the agonism of the GIP receptor is responsible for the observed results (45), particularly since the insulinotropic effect of GIP is known to be severely hampered in patients with T2D (12, 46).

More recently, the triple agonist of GLP-1, GIP, and glucagon receptors (GCGR), retatrutide, was also developed. Retatrutide has shown impressive WL results of up to 24.2% after 48 weeks of treatment (47), which appear to be even more substantial compared to tirzepatide, despite there are no head-to-head clinical trials comparing tirzepatide and retatrutide. The addition of GCGR agonism is hypothesized to lead to increased energy expenditure and decreased calorie intake, though the precise role of GCGR stimulation in retatrutide’s WL mechanism remains unclear (48).

In people with history of T2D before RYGB, we found no significant differences in the gut hormone profiles that were measured following a MMTT. Our results suggest that these hormone profiles do not seem to be influenced by T2D remission nor relapse following MBS. Thus, other mechanisms are likely to predominate at regulating glycemic status once excessive body weight is no longer in the equation.

Previous studies identified that preoperative insulin therapy, HbA1c levels and T2D duration are key factors in predicting T2D remission (49–51). In the current study, although none of the participants were on insulin therapy before surgery, the T2D relapse group exhibited higher preoperative HbA1c levels and longer T2D duration, suggesting a more pronounced β-cell dysfunction and reduced pancreatic reserve, which may have contributed to the T2D relapse.

Although not significant, our T2D relapse group did not lose as much % of body weight as our T2D remission group at nadir. While most studies suggest that the initial WL following RYGB is related with long-term T2D-related outcomes (16, 52, 53), a recent study found that WL had an impact on T2D remission in the first year, but did not influence the relapse rate in subsequent years (54). Taken together, these findings highlight stressing the importance of implementing dietary and lifestyle modifications following the surgical intervention in order to optimize the WL and consequent metabolic benefits, as early weight reduction may improve β-cell function and insulin sensitivity—both critical for achieving and sustaining T2D remission.

Although this study provides valuable insights, some limitations should be acknowledged. First, its cross-sectional design does not allow a confirmation of a causal relationships between hormone profiles and clinical surgical outcomes. The strict inclusion criteria for cohort 2 (patients with T2D relapse who were not receiving GLP-1 receptor agonists or insulin therapy; matched groups for pre-operative age, pre-operative BMI, time since surgery, and post-operative WL trajectories) were applied to avoid introducing bias into our results. Consequently, only 10 patients from our 10-year cohort qualified for the T2D relapse group. While this relatively small sample size, may limit the interpretation of the results, the detailed characterization of participants and the significant differences observed in their glucose and insulin profiles allowed us to investigate the impact of incretin hormones on these distinct metabolic profiles. Therefore, despite this limitation, the study provides valuable insights and supports the robustness of the findings. Additionally, although the type of RYGB procedure was not randomly assigned, the balanced distribution of surgical techniques reduces the potential for confounding. The influence of diabetes medications on meal test results, especially in cohort 2, should also be considered. To minimize this effect, only participants treated with metformin were included. Plasma glucose levels were measured using a glucometer, which has lower precision compared to laboratory-based plasma glucose measurements. However, since no part of the study protocol relied on real-time highly accurate glucose values, the method used for glucose measurement, although less precise, did not appear to raise significant concerns regarding the study’s overall findings.

In conclusion, we aimed to understand whether the enteropancreatic hormone profile of patients subjected to RYGB could be responsible for the long-term suboptimal response to the surgery, both regarding WR or T2D relapse. Although our results point towards a potential role of both GLP-1 and GIP in WR, this warrants further research. In contrast, the enteropancreatic hormone profile does not seem to be influenced by T2D remission nor relapse following BMS, and other mechanisms seem to be at play, including the natural history of disease progression. Overall, our results highlight a possible link between altered incretin responses and WR after RYGB, and support further prospective trials targeting GLP-1/GIP pathways to improve long-term metabolic outcomes.

## Data Availability

The raw data supporting the conclusions of this article will be made available by the authors, without undue reservation.

## References

[1] Adams TD Davidson LE Litwin SE Kim J Kolotkin RL Nanjee MN. Weight and metabolic outcomes 12 years after gastric bypass. New Engl J Med. (2017) 377:1143–55. doi: 10.1056/NEJMoa1700459 PMC5737957 28930514

[2] Courcoulas AP King WC Belle SH Berk P Flum DR Garcia L. Seven-year weight trajectories and health outcomes in the longitudinal assessment of bariatric surgery (Labs) study. JAMA Surg. (2018) 153:427–34. doi: 10.1001/jamasurg.2017.5025 PMC6584318 29214306

[3] Guimaraes M Osorio C Silva D Almeida RF Reis A Cardoso S. How sustained is roux-en-Y gastric bypass long-term efficacy?: roux-en-Y gastric bypass efficacy. Obes Surg. (2021) 31:3623–9. doi: 10.1007/s11695-021-05458-y PMC8270797 34021884

[4] Salminen P Kow L Aminian A Kaplan LM Nimeri A Prager G. Ifso consensus on definitions and clinical practice guidelines for obesity management-an international delphi study. Obes Surg. (2024) 34:30–42. doi: 10.1007/s11695-023-06913-8 37999891 PMC10781804

[5] Hindsø M Hedbäck N Svane MS Møller A Martinussen C Jørgensen NB. The importance of endogenously secreted glp-1 and gip for postprandial glucose tolerance and B-cell function after roux-en-Y gastric bypass and sleeve gastrectomy surgery. Diabetes. (2022) 72:336–47. doi: 10.2337/db22-0568 36478039

[6] Prasad M Mark V Ligon C Dutia R Nair N Shah A. Role of the Gut in the Temporal Changes of Beta-Cell Function after Gastric Bypass in Individuals with and without Diabetes Remission. Diabetes Care. (2022) 45:469–76. doi: 10.2337/dc21-1270 PMC8914419 34857533

[7] Jirapinyo P Jin DX Qazi T Mishra N Thompson CC. A meta-analysis of glp-1 after roux-en-Y gastric bypass: impact of surgical technique and measurement strategy. Obes Surg. (2018) 28:615–26. doi: 10.1007/s11695-017-2913-1 28871519

[8] le Roux CW Welbourn R Werling M Osborne A Kokkinos A Laurenius A. Gut hormones as mediators of appetite and weight loss after roux-en-Y gastric bypass. Ann Surg. (2007) 246:780–5. doi: 10.1097/SLA.0b013e3180caa3e3 17968169

[9] Davies M Faerch L Jeppesen OK Pakseresht A Pedersen SD Perreault L. Semaglutide 2.4 mg once a week in adults with overweight or obesity, and type 2 diabetes (Step 2): A randomised, double-blind, double-dummy, placebo-controlled, phase 3 trial. Lancet. (2021) 397:971–84. doi: 10.1016/S0140-6736(21)00213-0 33667417

[10] Pi-Sunyer X Astrup A Fujioka K Greenway F Halpern A Krempf M. A randomized, controlled trial of 3.0 mg of liraglutide in weight management. New Engl J Med. (2015) 373:11–22. doi: 10.1056/NEJMoa1411892 26132939

[11] Nogueiras R Nauck MA Tschop MH. Gut hormone co-agonists for the treatment of obesity: from bench to bedside. Nat Metab. (2023) 5:933–44. doi: 10.1038/s42255-023-00812-z 37308724

[12] Nauck MA Heimesaat MM Orskov C Holst JJ Ebert R Creutzfeldt W. Preserved incretin activity of glucagon-like peptide 1 [7–36 amide] but not of synthetic human gastric inhibitory polypeptide in patients with type-2 diabetes mellitus. J Clin Invest. (1993) 91:301–7. doi: 10.1172/JCI116186 PMC330027 8423228

[13] Del Prato S Gallwitz B Holst JJ Meier JJ. The incretin/glucagon system as a target for pharmacotherapy of obesity. Obes Rev. (2022) 23:e13372. doi: 10.1111/obr.13372 34713962 PMC9286339

[14] Nauck MA D'Alessio DA. Tirzepatide, a dual gip/glp-1 receptor co-agonist for the treatment of type 2 diabetes with unmatched effectiveness regrading glycaemic control and body weight reduction. Cardiovasc Diabetol. (2022) 21:169. doi: 10.1186/s12933-022-01604-7 36050763 PMC9438179

[15] Samms RJ Coghlan MP Sloop KW. How may gip enhance the therapeutic efficacy of glp-1? Trends Endocrinol Metab. (2020) 31:410–21. doi: 10.1016/j.tem.2020.02.006 32396843

[16] Cardoso S Pereira SS Almeida RF Osorio C Silva D Nora M. Accuracy of prediction models for long-term type 2 diabetes remission after gastric bypass. Acta Diabetol. (2023) 60:1019–26. doi: 10.1007/s00592-023-02092-1 PMC10289962 37085634

[17] Riddle MC Cefalu WT Evans PH Gerstein HC Nauck MA Oh WK. Consensus report: definition and interpretation of remission in type 2 diabetes. Diabetes Care. (2021) 44:2438–44. doi: 10.2337/dci21-0034 PMC8929179 34462270

[18] Nora M Guimaraes M Almeida R Martins P Goncalves G Freire MJ. Metabolic laparoscopic gastric bypass for obese patients with type 2 diabetes. Obes Surg. (2011) 21:1643–9. doi: 10.1007/s11695-011-0418-x 21512818

[19] Pereira AM Moura D Pereira SS Andrade S Almeida RF Nora M. Beyond restrictive: sleeve gastrectomy to single anastomosis duodenoileal bypass with sleeve gastrectomy as a spectrum of one single procedure. Obes Facts. (2024) 17(4):364–71. doi: 10.1159/000539104 PMC11299966 38801818

[20] Lobato CB Pereira SS Guimaraes M Hartmann B Wewer Albrechtsen NJ Hilsted L. A potential role for endogenous glucagon in preventing post-bariatric hypoglycemia. Front Endocrinol (Lausanne). (2020) 11:608248. doi: 10.3389/fendo.2020.608248 33424773 PMC7793799

[21] Hutch CR Sandoval D. The role of glp-1 in the metabolic success of bariatric surgery. Endocrinology. (2017) 158:4139–51. doi: 10.1210/en.2017-00564 PMC5711387 29040429

[22] Klein S Gastaldelli A Yki-Jarvinen H Scherer PE. Why does obesity cause diabetes? Cell Metab. (2022) 34:11–20. doi: 10.1016/j.cmet.2021.12.012 34986330 PMC8740746

[23] Gray SM Goonatilleke E Emrick MA Becker JO Hoofnagle AN Stefanovski D. High doses of exogenous glucagon stimulate insulin secretion and reduce insulin clearance in healthy humans. Diabetes. (2024) 73:412–25. doi: 10.2337/db23-0201 PMC10882148 38015721

[24] Svendsen B Larsen O Gabe MBN Christiansen CB Rosenkilde MM Drucker DJ. Insulin secretion depends on intra-islet glucagon signaling. Cell Rep. (2018) 25:1127–34.e2. doi: 10.1016/j.celrep.2018.10.018 30380405

[25] den Biggelaar LJ Sep SJ Eussen SJ Mari A Ferrannini E van Greevenbroek MM. Discriminatory ability of simple ogtt-based beta cell function indices for prediction of prediabetes and type 2 diabetes: the codam study. Diabetologia. (2017) 60:432–41. doi: 10.1007/s00125-016-4165-3 PMC6518926 27933333

[26] Utzschneider KM Prigeon RL Faulenbach MV Tong J Carr DB Boyko EJ. Oral disposition index predicts the development of future diabetes above and beyond fasting and 2-H glucose levels. Diabetes Care. (2009) 32:335–41. doi: 10.2337/dc08-1478 PMC2628704 18957530

[27] Sugiyama S Jinnouchi H Hieshima K Kurinami N Jinnouchi K Yoshida A. Potential identification of type 2 diabetes with elevated insulin clearance. NEJM Evid. (2022) 1:EVIDoa2100052. doi: 10.1056/EVIDoa2100052 38319210

[28] Moffett RC Docherty NG le Roux CW. The altered enteroendocrine reportoire following roux-en-Y-gastric bypass as an effector of weight loss and improved glycaemic control. Appetite. (2021) 156:104807. doi: 10.1016/j.appet.2020.104807 32871202

[29] Dirksen C Jørgensen NB Bojsen-Møller KN Kielgast U Jacobsen SH Clausen TR. Gut hormones, early dumping and resting energy expenditure in patients with good and poor weight loss response after roux-en-Y gastric bypass. Int J Obes (Lond). (2013) 37:1452–9. doi: 10.1038/ijo.2013.15 23419600

[30] Çalık Başaran N Dotan I Dicker D. Post metabolic bariatric surgery weight regain: the importance of glp-1 levels. Int J Obes. (2025) 49:412–7. doi: 10.1038/s41366-024-01461-2 PMC11971041 38225284

[31] Sima E Webb DL Hellström PM Sundbom M. Non-responders after gastric bypass surgery for morbid obesity: peptide hormones and glucose homeostasis. Obes Surg. (2019) 29:4008–17. doi: 10.1007/s11695-019-04089-8 31338735

[32] Santo MA Riccioppo D Pajecki D Kawamoto F de Cleva R Antonangelo L. Weight regain after gastric bypass: influence of gut hormones. Obes Surg. (2016) 26:919–25. doi: 10.1007/s11695-015-1908-z 26450709

[33] Shantavasinkul PC Omotosho P Muehlbauer MJ Natoli M Corsino L Tong J. Metabolic profiles, energy expenditures, and body compositions of the weight regain versus sustained weight loss patients who underwent roux-en-Y gastric bypass. Surg Obes Relat Dis. (2021) 17:2015–25. doi: 10.1016/j.soard.2021.09.007 34635422

[34] Lampropoulos C Mulita F Alexandrides T Kehagias D Kalavrizioti D Albanopoulos K. Ghrelin, Glucagon-Like Peptide-1, and Peptide Yy Secretion in Patients with and without Weight Regain during Long-Term Follow-up after Bariatric Surgery: A Cross-Sectional Study. Prz Menopauzalny. (2022) 21:97–105. doi: 10.5114/pm.2022.116492 36199737 PMC9528819

[35] Gao Z Yang J Liang Y Yang S Zhang T Gong Z. Changes in gastric inhibitory polypeptide (Gip) after roux-en-Y gastric bypass in obese patients: A meta-analysis. Obes Surg. (2022) 32:2706–16. doi: 10.1007/s11695-022-05959-4 35597875

[36] van Bloemendaal L ten Kulve JS la Fleur SE Ijzerman RG Diamant M. Effects of glucagon-like peptide 1 on appetite and body weight: focus on the cns. J Endocrinol. (2014) 221:T1–T16. doi: 10.1530/joe-13-0414 24323912

[37] Thondam SK Cuthbertson DJ Wilding JPH. The influence of glucose-dependent insulinotropic polypeptide (Gip) on human adipose tissue and fat metabolism: implications for obesity, type 2 diabetes and non-alcoholic fatty liver disease (Nafld). Peptides. (2020) 125:170208. doi: 10.1016/j.peptides.2019.170208 31759125

[38] Lean ME Malkova D. Altered gut and adipose tissue hormones in overweight and obese individuals: cause or consequence? Int J Obes (Lond). (2016) 40:622–32. doi: 10.1038/ijo.2015.220 PMC4827002 26499438

[39] Rao RS Kini S. Gip and bariatric surgery. Obes Surg. (2011) 21:244–52. doi: 10.1007/s11695-010-0305-x 21082290

[40] Vilsboll T Krarup T Sonne J Madsbad S Volund A Juul AG. Incretin secretion in relation to meal size and body weight in healthy subjects and people with type 1 and type 2 diabetes mellitus. J Clin Endocrinol Metab. (2003) 88:2706–13. doi: 10.1210/jc.2002-021873 12788877

[41] Rijkelijkhuizen JM McQuarrie K Girman CJ Stein PP Mari A Holst JJ. Effects of meal size and composition on incretin, alpha-cell, and beta-cell responses. Metabolism. (2010) 59:502–11. doi: 10.1016/j.metabol.2009.07.039 19846181

[42] Nauck MA Müller TD. Incretin hormones and type 2 diabetes. Diabetologia. (2023) 66:1780–95. doi: 10.1007/s00125-023-05956-x PMC10474001 37430117

[43] Al-Massadi O Fernø J Diéguez C Nogueiras R Quiñones M. Glucagon control on food intake and energy balance. Int J Mol Sci. (2019) 20(16):3905. doi: 10.3390/ijms20163905 31405212 PMC6719123

[44] Pérez-Arana GM Díaz-Gómez A Bancalero-de Los Reyes J Gracia-Romero M Ribelles-García A Visiedo F. The role of glucagon after bariatric/metabolic surgery: much more than an "Anti-insulin" Hormone. Front Endocrinol (Lausanne). (2023) 14:1236103. doi: 10.3389/fendo.2023.1236103 37635984 PMC10451081

[45] Willard FS Douros JD Gabe MB Showalter AD Wainscott DB Suter TM. Tirzepatide is an imbalanced and biased dual gip and glp-1 receptor agonist. JCI Insight. (2020) 5(17):e140532. doi: 10.1172/jci.insight.140532 32730231 PMC7526454

[46] Vilsboll T Knop FK Krarup T Johansen A Madsbad S Larsen S. The pathophysiology of diabetes involves a defective amplification of the late-phase insulin response to glucose by glucose-dependent insulinotropic polypeptide-regardless of etiology and phenotype. J Clin Endocrinol Metab. (2003) 88:4897–903. doi: 10.1210/jc.2003-030738 14557471

[47] Jastreboff AM Kaplan LM Frías JP Wu Q Du Y Gurbuz S. Triple-hormone-receptor agonist retatrutide for obesity - a phase 2 trial. New Engl J Med. (2023) 389:514–26. doi: 10.1056/NEJMoa2301972 37366315

[48] Abdul-Rahman T Roy P Ahmed FK Mueller-Gomez JL Sarkar S Garg N. The power of three: retatrutide's role in modern obesity and diabetes therapy. Eur J Pharmacol. (2024) 985:177095. doi: 10.1016/j.ejphar.2024.177095 39515565

[49] Capoccia D Leonetti F Natali A Tricò D Perrini S Sbraccia P. Remission of type 2 diabetes: position statement of the italian society of diabetes (Sid). Acta Diabetologica. (2024) 61:1309–26. doi: 10.1007/s00592-024-02317-x PMC11486812 38942960

[50] Hariri K Guevara D Jayaram A Kini SU Herron DM Fernandez-Ranvier G. Preoperative insulin therapy as a marker for type 2 diabetes remission in obese patients after bariatric surgery. Surg Obes Relat Dis. (2018) 14:332–7. doi: 10.1016/j.soard.2017.11.016 29339030

[51] Debédat J Sokolovska N Coupaye M Panunzi S Chakaroun R Genser L. Long-term relapse of type 2 diabetes after roux-en-Y gastric bypass: prediction and clinical relevance. Diabetes Care. (2018) 41:2086–95. doi: 10.2337/dc18-0567 30082327

[52] Pucci A Tymoszuk U Cheung WH Makaronidis JM Scholes S Tharakan G. Type 2 diabetes remission 2 years post roux-en-Y gastric bypass and sleeve gastrectomy: the role of the weight loss and comparison of diarem and diabetter scores. Diabetes Med. (2018) 35:360–7. doi: 10.1111/dme.13532 PMC5836992 29055156

[53] Sjöström L Peltonen M Jacobson P Ahlin S Andersson-Assarsson J Anveden Å. Association of bariatric surgery with long-term remission of type 2 diabetes and with microvascular and macrovascular complications. JAMA. (2014) 311:2297–304. doi: 10.1001/jama.2014.5988 24915261

[54] Moriconi D Manca ML Anselmino M Rebelos E Bellini R Taddei S. Predictors of type 2 diabetes relapse after roux-en-Y gastric bypass: A ten-year follow-up study. Diabetes Metab. (2022) 48:101282. doi: 10.1016/j.diabet.2021.101282 34547450

