# Supplementary material for: GLP-1 and GIP may play a role in long-term weight trajectories after gastric bypass
Source: Front Endocrinol (Lausanne). 2025 Jul 4;16:1624001. doi: 10.3389/fendo.2025.1624001 (PMC12271881; doi:10.3389/fendo.2025.1624001)
Supplement: Supplementary file 1 [file DataSheet1.docx]

Supplementary Material


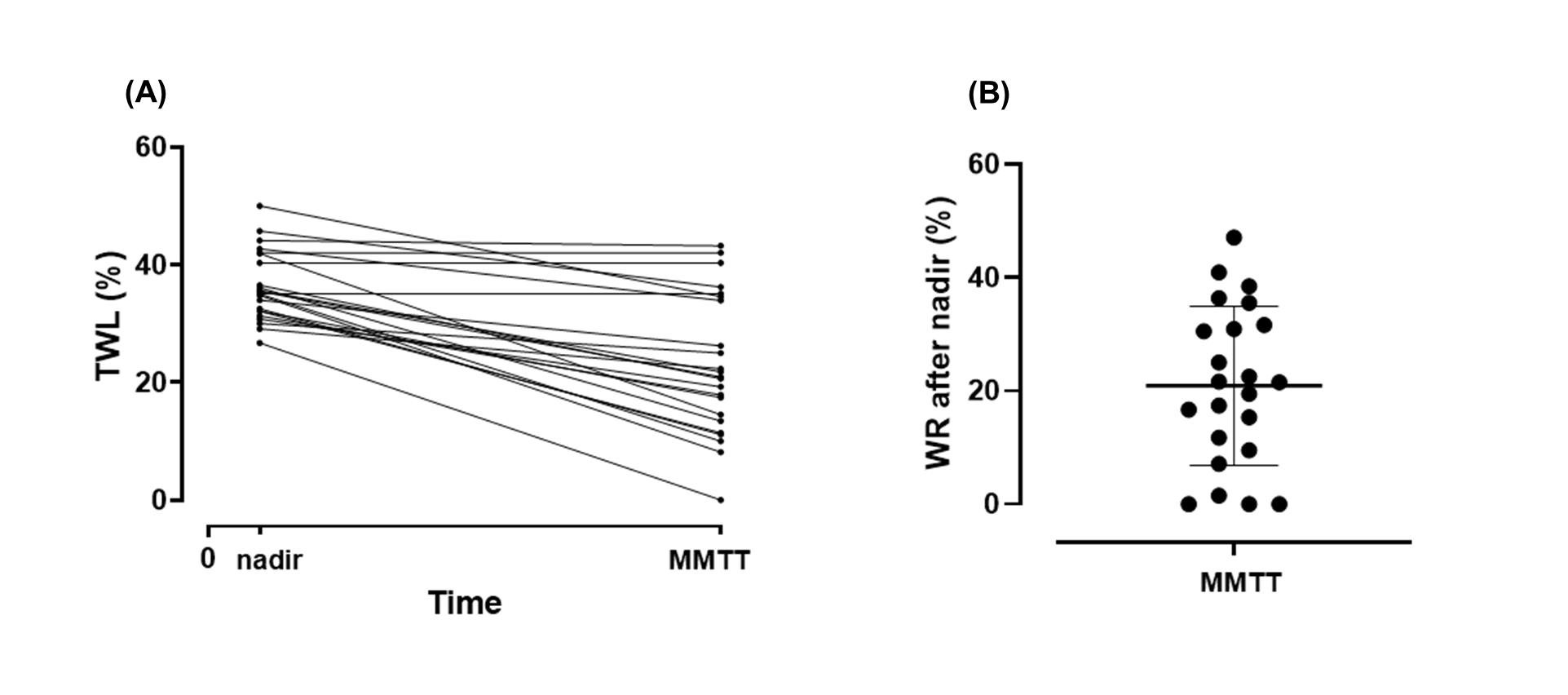


**Supplementary Figure 1.** Percentage of total weight loss (%TWL) at the nadir and at the mixed meal tolerance test (MMTT) timepoints (A), and weight regain (%WR) after the nadir at the MMTT timepoint (B). Only patients with no Type 2 diabetes history were included (Cohort 1, n=23)

**Supplementary Table 2.** Impact of participants age, body weight and BMI before surgery on the % of total weight loss and weight regain after nadir until the MMTT

|  | %TWL | %WR |
| --- | --- | --- |
| Age (years) | β=-0.60 (-1.42; 0.21)  p=0.137 | β=1.40 (-1.57; 4.39)  p=0.335 |
| Body weight (kg) | **β=-3.88 (-0.74; -0.03)**  **p=0.034** | **β=1.73 (0.55; 2.90)**  **p=0.006** |
| BMI (kg/m^2^) | **β=-1.71 (-2.95; -0.46)**  **p=0.009** | **β=6.02 (1.47; 10.6)**  **p=0.012** |

*BMI* body mass index, *TWL* total weight loss, *MMTT* mixed meal tolerance test, *WR* weight regain. Only patients with no Type 2 diabetes history were included (Cohort 1, n=23). Linear regression analysis: *statistically significant differences (p<0.05) are highlighted in bold.*
